# Plastibell circumcision of 2,276 male infants: a multi-centre study

**DOI:** 10.11604/pamj.2016.23.35.7841

**Published:** 2016-02-09

**Authors:** Bioku Muftau Jimoh, Ikuerowo Stephen Odunayo, Igwilo Chinwe, Omisanjo Olufunmilade Akinfolarin, Adewumi Oluwafemi, Esho Julius Olusanmi

**Affiliations:** 1 Department of Surgery, Federal Staff Medical Centre, Abuja; 2 Urology Unit, Department of Surgery, Lagos State University Teaching Hospital, Ikeja; 3 Department of Obstetrics and Gynecology, Federal Staff Medical Centre, Abuja

**Keywords:** Plastibell, Circumcision, anesthesia, bleeding

## Abstract

**Introduction:**

The World Health Organization's manual on male circumcision listed Plastibell technique as a well-proven paediatric method with respect to the results and complications. Although, literatures abound on its wide acceptability, there are few multi-centered reports from this environment. The objective was to evaluate the cases of infant circumcision by Plastibell device from two medical institutions.

**Methods:**

All consecutive infants who had Classical Plastibell Circumcision (PC) at the Federal Staff Medical Centre, Abuja and the Lagos State University Teaching Hospital, Ikeja between February 2011 and June 2015 were included in this cross-sectional study. The procedures were performed by surgical registrars and medical officers after ninety minutes of topical anesthesia to the penis. Data harvested from the standard proforma were analysed using Statistical Package for Social Science 20.0 for window.

**Results:**

A total of 2,276 infants had classical PC within the study period. Their ages at circumcision ranged from 4 days to 3 months with a mean age of 17 days. Majority of the boys were circumcised at second week of life (n=1,394,61.2%). All the cases were performed for religious (53%) and cultural (47%)reasons. The most common Plastibell size deployed was 1.3cm (n=1,040, 45.7%) while 1.6cm was the least commonly used ring (n=10, 0.4%). The mean time for device to fall-off was 6 days (range 4-12 days). There was no correlation between the age at circumcision and Plastibell size. We recorded an overall complication rate of 1.1% with postoperative bleeding leading the pack (n=12, 48%). No case of urethrocutaneous fistula was seen. We detected 17 cases (0.7%) of distal hypospadias in whom circumcisions were postponed till the time of hypospadias repairs.

**Conclusion:**

The main indication for infant circumcision in our environment was religious. The PC has good safety profile with few easily correctable early complications. Detailed attention to placement of ligature, selection of appropriate Plastibell size and adequate parental education are key to preventing post-procedure mishaps.

## Introduction

Male circumcision connotes surgical removal of prepuce that covers the glans penis. It remains one of the most common operations performed globally [1] for therapeutic, prophylactic, religious, cultural or social reasons. Demographic and health surveys ranked Nigeria among the high male circumcision prevalence nations [2–4]. None-the-less, the exact percentage is unknown. Hitherto, conventional dissection surgeries were deployed by both orthodox and non-orthodox circumcisers. Recently however, these procedures are carried out using plastibell device [5–8]. Since it was first reported in 1956 [9], Plastibell circumcision (PC) has gained widespread use [10–13]. The World Health Organization (WHO)'s manual on male circumcision listed the technique as a well proven method with respect to its results and complications. Plastibell device is a clear plastic ring with handle. The ring, which comes in different sizes, has a deep groove running circumferentially. A cotton thread is usually included in the pack. In order to reduce complications such as bleeding in its classical form, some modifications have been made [13]. Plastibell circumcision is safe and easy to perform especially in infants using only local anesthesia with very few associated mishaps [3, 7, 8]. The other devices such as Gomgo clamp and Mogen device are rarely used in our setting. There is avalanche of literatures on the wide acceptability of male circumcision by plastibell device [8–10, 14, 15] but few multi-centered reports from this environment exist. The aim of this study was to evaluate cases of infant circumcision by plastibell devices from two medical institutions. Its ease of performance and safety profile were re-echoed.

## Methods

All consecutive infants who had classical PC at the Federal Staff Medical Centre, Abuja and the Lagos State University Teaching Hospital, Ikeja between February 2011 and June 2015 were included in this cross-sectional study. A standard proforma was employed as approved by the hospital's ethical committee and all parents of the subjects signed informed consent. The data obtained regarding the age of the child, reason(s) for the circumcision, the surgeon, mode of anesthesia, plastibell size, incidental congenital penile abnormality, time taken for the device to fall-off and complication(s) were analysed using Statistical Package for Social Sciences 20.0 for window. **Anesthesia:** eutectic Mixture of Local Anesthetics (EMLA) 5% cream (containing 2.5% lidocaine and 2.5% prilocaine) was applied to the whole penis approximately 90 minutes before the procedure. **Procedure:** the procedures were performed in the operating suite by Medical officer or Surgical resident. With the patient in supine position and restrained by a nurse, the penile shaft was prepped with cetrimide. The prepuce was separated from the glans by using a blunt curved mosquito artery forceps and gauze. The dorsum of the foreskin was slamped at 12 O'clock for ten seconds and slit until corona was visible. The appropriate size plastibell was then positioned over the glans and prepuce brought over it. This was secured with a cotton thread supplied with the device. The prepuce was then trimmed and handle of the ring snapped. The site was inspected for any bleeding, meatal opening and correct positioning of the ligature. All the children were sent home on oral antibiotics and analgesics. Parents were given specific instructions on care of the device and were contacted on mobile phones as appropriate.

## Results

**Figure 1:**
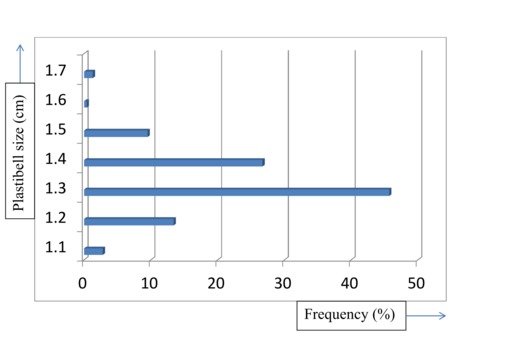
Plastibell size deployed

**Figure 2:**
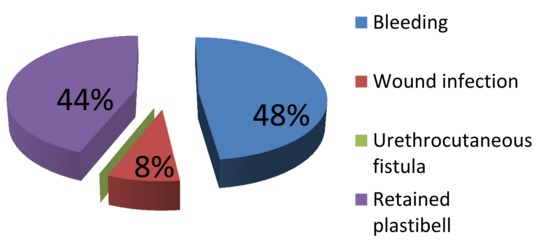
Immediate post-circumcision complications

A total of 2,276 infants had Classical PC within the study period. Their ages ranged from 4 days to 3 months with a mean age of 17 days. Majority of the boys were circumcised at second week of life (n=1,394, 61.2%) as detailed in Table 1. All the procedures were carried out under topical EMLA 5% cream. The indications for circumcision were mainly religious (53%) and cultural (47%). We found out that the most common plastibell size deployed was 1.3cm (n=1,040, 45.7%) while 1.6cm was the least commonly used ring (n=10, 0.4%) as seen in Figure 1. However there was no correlation between the age at circumcision and the plastibell size. The mean time for device to fall-off was 6 days (range 4-12 days). Figure 2 depicts the immediate complications of the procedure. We recorded an overall complication rate of 1.1% (n=25) with postoperative bleeding leading the pack (n=12, 48%). Some of the bleeding cases (n=7,58%) resolved with local compression and tightening of ligature while the others (n=5,42%) were converted to dissection technique. No case of urethrocutaneous fistula was seen. Our study detected 17 cases (0.7%) of distal hypospadias. These children were later circumcised during hypospadias repair.

**Table 1:** Age distribution of circumcised patients

Age (Days)	Frequency	%
1-7	152	6.7
8-14	1,394	61.2
15-21	428	18.8
22-28	83	3.6
>28	218	9.6
Total	2,276	100

## Discussion

Nigeria is ranked among the male circumcision prevalence nations [2–4, 16] with plastibell method gaining wide acceptability [14, 15]. Circumcision with plastibell is a safe and simple procedure especially in trained hands and for children below 2 years of age [14]. In this study, we applied topical EMLA 5% cream to the penis approximately 90 minutes prior to the procedure for all patients. But the effectiveness of this agent was not assessed. However, it has been documented that the topical EMLA provides effective anesthesia during circumcision [17, 18] but is less so than ring block or dorsal penile block or combination of both [17–20]. Other modes of anesthesia administered singly or in combination include pacifier (especially glucose or sucrose) [21], music [22] and tetracaine gel [23]. Anesthesia is recommended by WHO for pediatric circumcision as many studies have shown that babies react to pain [24, 25]. There is regional as well as religious variation in typical age of circumcision [8, 26–28]. We found out that majority of the boys were circumcised at second week of life (61.2%). This finding may be adduced to the study settings (Abuja and Lagos) where preponderance of our patients were Yorubas and Igbos. The Igbos are mainly Christians [8] while the Yorubas often circumcised their boys around 7th day of life so as to coincide with the naming ceremony [24, 29]. Male circumcision has been widely practiced for therapeutic, prophylactic, religious, cultural or social reasons [30–32]. In our study, all the plastibell circumcisions were carried out for religious and cultural reasons.

**Figure 3:**
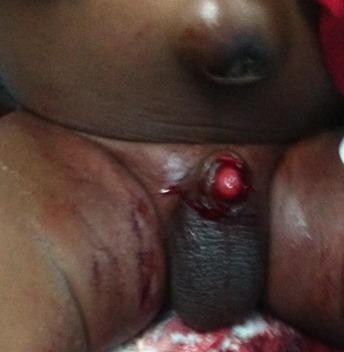
Post-circumcision bleeding

**Figure 4:**
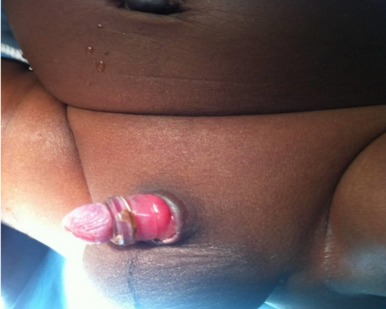
Retained plastibell ring

**Figure 5:**
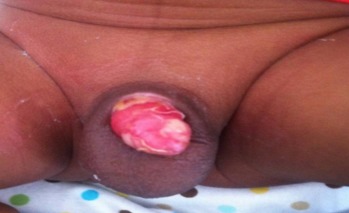
Post-circumcision wound infection

The most common plastibell size deployed was 1.3cm, similar to findings in other series [29]. But this is in contrast to a Jos, Nigeria study [7] where 1.2cm was the modal size. The plastibell comes in 7 sizes (1.1cm to1.7cm). An appropriate size is selected by visual estimate of the glans girth which gets better with practice and experience. This is important as undersized and oversized rings may result in tissue necrosis as well as retained plastibell and proximal ring migration respectively [5, 14, 33]. Most of the researches on PC are related to its complications [5, 6, 11, 33, 34]. These are usually minor and easily treatable, and include bleeding, retained plastibell and wound infection. Others are skin bridge, meatal stenosis, ring migration and urethrocutaneous fistula [35]. Death following the procedure has been reported [15, 36, 37]. We recorded an overall complication rate of 1.1% with bleeding topping the list (n=12, 48%) (Figure 3). This is corroborated by other works [7, 33]. The low frequency of our post-operative complications may be because the procedures were performed by medically trained providers. The identified causes of bleeding were loose ligature (n=5, 42%), glanular abrasion (n=2, 16%) and tearing of frenular vessels (n=5, 42%). Detailed attention to ensure ligature is tightly secured [7, 33]and use of modified plastibell ring that easily settle down circumferentially at corona and fitted well over the frenular fold are key to preventing post-circumcision bleeding [7, 13, 33]. Retained plastibell ring was the second most common complication recorded. These were noted in 11cases (0.48%) (Figure 4). They were undersized rings which got impacted on the glans and were easily removed by either loosening the ligature or using ring-cutter. Researchers have shown that plastibell rings usually fall-off within 10 days of the procedure. It has also been demonstrated that the ring separates faster in younger children due to thin prepuce and easier sloughing [7, 38]. We encountered wound infection in (0.1%) of cases (Figure 5). Meticulous attention to aseptic technique and routine use of prophylactic oral antibiotics postoperatively is probably responsibly for lower rate of infection compared to that reported in other study [13].

## Conclusion

The main indication for infant circumcision in our environment was religious. The classical PC has good safety profile with few easily correctable early complications. Detailed attention to placement of ligature, selection of appropriate Plastibell size and adequate parental education are relevant to preventing post-procedure mishaps.

### What is known about this topic

- Male infant circumcision by Plastibell device is widely acceptable worldwide with diverse indications
- Plastibell circumcision is safe and easy to perform

### What this study adds

- We found out that the main indication for infant circumcision in our environment was religious.
- The study reinforced the need to pay detailed attention to selection of appropriate Plastibell size, placement of ligature and parental education in reducing post-procedure complications.
